# Hemodynamic impact of surgical correction of pectus excavatum - a cardiovascular magnetic resonance study

**DOI:** 10.1186/1532-429X-13-S1-P190

**Published:** 2011-02-02

**Authors:** Anja Zagrosek, Florian von Knobelsdorff-Brenkenhoff, Susanne Polleichtner, Klaus Schaarschmidt, Jeanette Schulz-Menger

**Affiliations:** 1 HELIOS Klinikum Berlin-Buch, Charité Campus-Buch, Humboldt University, Berlin, Germany; 2 HELIOS Klinikum Berlin-Buch, Klinik für Kinderchirurgie, Berlin, Germany

## Objectives

The aim of the present study was to evaluate the impact of pectus excavatum-surgery on cardiac function with the use of cardiovascular magnetic resonance (CMR).

## Background

It is unclear, whether the surgical repair of pectus excavatum (PE) improves cardiovascular function or simply remains a cosmetic procedure. Previous studies with small sample sizes provided conflicting results. Therefore, health insurances currently refuse to pay for this intervention.

## Methods

**Figure 1:**
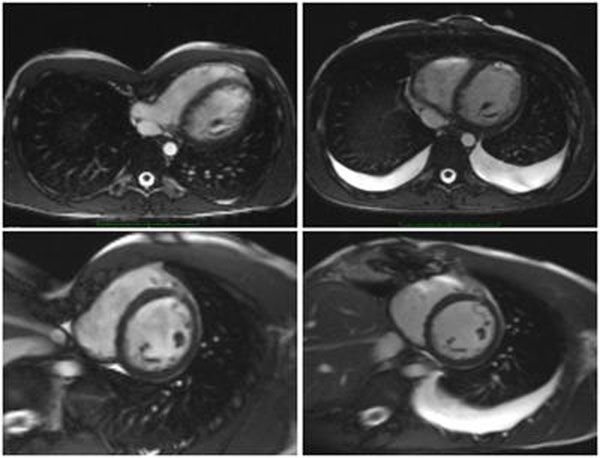
Upper row: Axial SSFP-images before (left) and after (right) surgery Lower row: Short axis SSFP-images before (left) and after (right) surgery.

28 patients (age [mean±standard deviation] 21.1±8.6 years, 5 female, BMI 20.8±3.6 kg/m2) scheduled for surgical correction of PE underwent CMR in a 1.5 T Scanner before and 9.7±1.4 days after surgery (Nuss procedure: implantation of two transthoracical pectus bars). 17 patients returned for a follow up-CMR 95.8±20.2 days after surgery. Cardiac dimensions and function were assessed with cine-SSFP-imaging in short axis orientation for the left ventricle (LV) and for the right ventricle (RV) in axial orientation (imaging parameters: repetition time 2.9 ms; echo time 1.2 ms; flip angle 80°; field of view 340 to 380 mm2; matrix 256 × 146; bandwidth 930 Hz/px; 30 phases per R-R-interval, for LV: slice thickness 7 mm; gap 3 mm, and for RV: slice thickness 5 mm; no gap). Phase contrast CMR (repetition time 38.9 ms; echo time 2.8 ms; flip angle 30°; field of view 219 x 319 mm; matrix 132 x 192; bandwidth 375 Hz/px; slice thickness 5.5 mm) was used to assess RV- and LV stroke volumes (SV). Figure 1

## Results

LV- and RV-ejection fraction increased significantly after surgery as well as at follow-up. LV-enddiastolic volume (LV-EDV) remained unchanged, whereas the RV-EDV decreased significantly after surgery. There was a tendency towards increased LV- and RV-stroke volumes after surgery and at follow up and towards a concurrently decreased heart rate (see table 1 for detailed results). All patients had bilateral pleural effusion after the intervention, which resolved almost entirely at follow up.

**Table 1:** CMR parameters before and after surgery and at follow-up

	Pre-op	Post-op	Pre-op vs. post-op P=	Follow-up (n=17)	Pre-op vs. follow-up P=
LV-ejection fraction (%)	59.0±5.1	62.5±3.4	<0.001	61.3±3.7	<0.001
RV-ejection fraction (%)	47.0±5.3	51.3±5.9	<0.001	50.1±3.9	<0.001
LV-EDV (ml)	158.5±32.5	161.2±34.1	ns	155.4±30.1	ns
RV-EDV (ml)	203.6±38.7	195.3±41.9	0.047	189.1±32.9	<0.001
LV-SV (ml)	86.0±16.6	92.4±15.0	0.006	89.7±14.8	ns
RV-SV (ml)	90.6±18.8	99.9±20.5	0.001	93.9±13.7	ns
Heart rate (bpm)	77.1±12.3	76.6±12.9	ns	72.3±11.8	ns

legends: ns: not significant, LV: left ventricular, RV: right ventricular, EDV: enddiastolic volume, SV: stroke volume, bpm: beats per minute.

## Conclusion

The findings of the present study suggest that surgical repair of pectus excavatum leads to changes in cardiac dimensions and function, contradicting the argument that this procedure is primarily cosmetic. Further studies with larger samples with inclusion of exercise tests are required.

